# Spatial Pattern of Land Use Change and Its Driving Force in Jiangsu Province

**DOI:** 10.3390/ijerph110303215

**Published:** 2014-03-18

**Authors:** Xindong Du, Xiaobin Jin, Xilian Yang, Xuhong Yang, Yinkang Zhou

**Affiliations:** School of Geographic and Oceanographic Sciences, Nanjing University, Nanjing 210093, China; E-Mails: dxd1008@163.com (X.D.); psdm@smail.nju.edu.cn (X.Y.); mg1030037@smail.nju.edu.cn (X.Y.); 1963hp@sohu.com (Y.Z.)

**Keywords:** spatial pattern, land use change, driving force, Jiangsu Province, China

## Abstract

Scientific interpretation of the mechanism of land use change is important for government planning and management activities. This study analyzes the land use change in Jiangsu Province using three land use maps of 2000, 2005 and 2008. The study results show that there was a significant change in land use. The change was mainly characterized by a continuous built-up land expansion primarily at the expense of cropland loss, and the trend became increasingly rapid. There was an obvious regional difference, as most of the cropland loss or built-up land expansion took place in southern Jiangsu, where the rate of built-up land expansion was faster than in central and northern Jiangsu. Meanwhile, the spatial pattern changed remarkably; in general, the number of patches (NumP) showed a declining trend, and the mean patch size (MPS) and patch size standard deviation (PSSD) displayed increase trends. Furthermore, the relative importance of selected driven factors was identified by principal component analysis (PCA) and general linear model (GLM). The results showed that not only the relative importance of a specific driving factor may vary, but the driven factors may as well. The most important driven factor changed from urban population (UP), secondary gross domestic product (SGDP) and gross domestic product (GDP) during 2000–2005 to resident population (RP), population density (POD) and UP during 2005–2008, and the deviance explained (DE) decreased from 91.60% to 81.04%. Policies also had significant impacts on land use change, which can be divided into direct and indirect impacts. Development policies usually had indirect impacts, particularly economic development policies, which promote the economic development to cause land use change, while land management policies had direct impacts. We suggest that the government should think comprehensively and cautiously when proposing a new development strategy or plan.

## 1. Introduction

Land-use/land-cover change (LUCC) may significantly affect the ecological environment and sustainable development [1,2]. LUCC is receiving an increasing amount of attention from scholars, and there were numerous studies on LUCC have been reported around the world at various scales [3,4,5,6,7,8,9,10,11,12]. So far the research contents mainly included described land use change [13,14], explored the relationship between LUCC and driving forces [15], simulated land use change [16,17], and analyzed the impacts of land use change [18]. Meanwhile, developing countries are undergoing rapid urbanization and industrialization, which leads to more rapid LUCC than in developed countries [11]. Over the past several decades, China has been the World’s most populous country, as well as one of the most rapidly developing countries, and along with its continuous urbanization and population increase, China has been undergoing an increasingly significant landscape changes [19,20,21,22,23,24,25]. Most previous studies have focused on a city or basin-scale [26,27,28,29,30,31,32,33,34], and little research has been done to assess the land-use change at a provincial level. Due to the special political characteristics of China, provincial governments have more power to propose specific rules and regulations or policy evaluation criteria; for example, the conversion of cropland to built-up land must be approved by the provincial-level government or higher [35]. It means that explore the LUCC at a provincial level may provide more valuable information for the government’s planning and management activities. A few studies have been done to assess the provincial LUCC and its driving mechanisms in areas such as Heilongjiang [36], Beijing [37], Sichuan [38,39], Jiangxi [40], and so on. However, most of these studies did not identify the relative importance of driving forces, and almost all studies assumed that the importance of each driving factor was not change during the whole study period. In this paper, we analyze the land use change in Jiangsu Province from 2000 to 2008, and identify the importance of the social-economic driving force in the periods from 2000 to 2005 and 2005 to 2008. In addition, Jiangsu Province is one of the most highly developed regions in China, and the landscape pattern has experienced great changes, so the study results of this paper may provide a reference for other less developed provinces [41].

## 2. Study Area and Methodology

### 2.1. Study Area

Jiangsu Province consists of 13 prefecture-level cities, namely Nanjing (NJ), Wuxi (WX), Changzhou (CZ), Suzhou (SZ), Zhenjing (ZJ), Nantong (NT), Yangzhou (YZ), Taizhou (TZ), Xuzhou (XZ), Lianyungang (LYG), Huai’an (HA), Yancheng (YC) and Suqian (SQ). Nanjing is the capital of Jiangsu Province, and Suzhou is the most highly developed city. Jiangsu Province is usually divided into three areas according to location and economics; these three areas are southern Jiangsu, including Nanjing, Wuxi, Changzhou, Suzhou and Zhenjing; central Jiangsu, including Nantong, Yangzhou and Taizhou; and northern Jiangsu, including Xuzhou, Lianyungang, Huai’an, Yancheng and Suqian. There is a significant economic gap among the three areas, the regional difference in 2008 being about 3.16:1.73:1, which is similar to the gap between eastern China, central China and western China. In order to respond to some of the central government’s policies, the Jiangsu provincial government implemented many regional polices to promote the region’s balanced development and economic growth of undeveloped areas; one of these policies was that the industry area was to be transferred from southern Jiangsu to central and northern Jiangsu. These policies had also played an important role in social development, and some policies may affect the land use decisions. Due to these factors, we choose Jiangsu Province as the study area, and quantified the LUCC change and spatial patterns, which may improve our understanding of the mechanism of grain yield change, in turn aiding a more effective regional planning and natural resource management.

**Figure 1:**
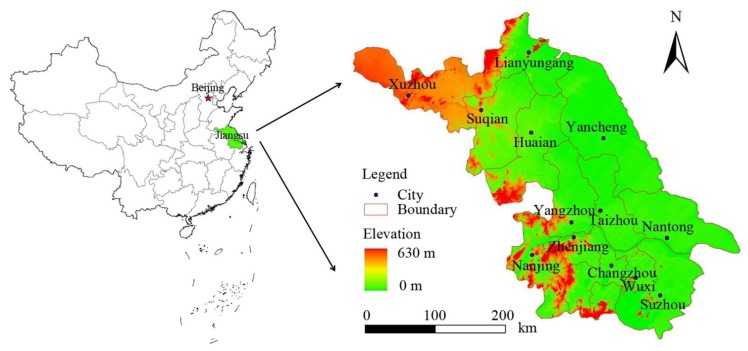
Location of the study area.

Jiangsu Province is one of most prosperous regions in China, and is located in eastern coastal China between longitudes 116°18′ E and 121°57′ E, and latitudes 30°45′ N and 35°20′ N (Figure 1). This region is characterized by an eastern Asian monsoon climate with an annual temperature of approximately 13.6–16.1 °C, and annual rainfall of approximately 1,000 mm. Topographically, the main terrain is plain, which accounts for 85% of the study area, and hilly terrain accounts for 15%, with elevation ranging from 0 to 625 m. Benefiting from excellent natural conditions, Jiangsu Province has become one of China’s most important grain production bases. 

Jiangsu Province had a population of 74.38 million in 2000 and 78.66 million in 2010, with an annual growth rate of about 0.56%. Since the implementation of China’s policy of reform and opening to the world in 1978, Jiangsu Province has experienced a variety of social-economic changes, with the GDP *per capita* increasing from 11,765 to 40,014 yuan, this was significantly above the national average of 23,677 yuan. Along with the increase of population and economic development, LUCC has been undergoing a constant change, which has led to environmental and ecological problems such as water pollution, land degradation, soil contamination and biodiversity loss [42,43,44,45,46,47,48,49,50,51,52,53,54,55].

### 2.2. Data Source

**Figure 2:**
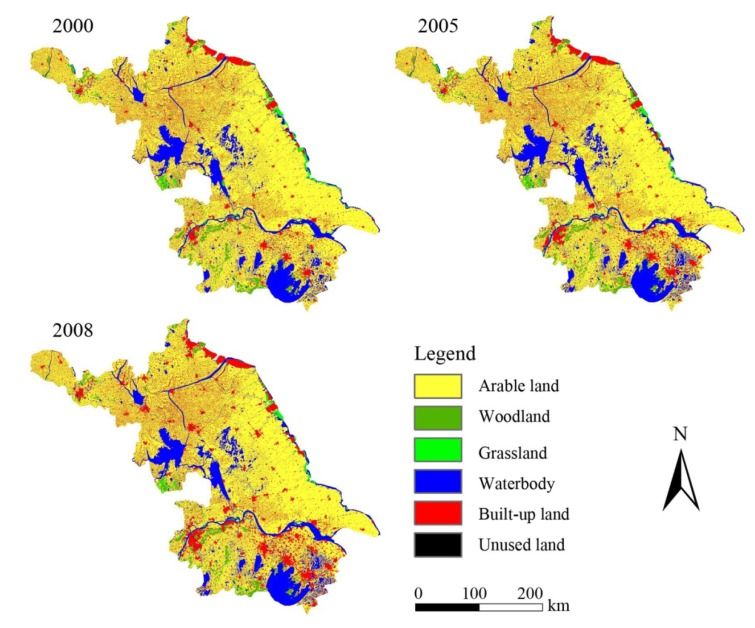
Land use map of Jiangsu Province during 2000, 2005 and 2008.

In this paper, we used three land-use maps from 2000, 2005 and 2008, respectively. The datasets were obtained from the Data Center for Resources and Environmental Sciences Chinese Academy of Sciences (RESDC), which were produced by the Chinese Academy of Sciences at a scale of 1:100,000, with six primary levels and 25 secondary levels of land-use classes. These maps were produced using satellite images, such as Landsat TM/ETM, China-Brazil Earth Resources Satellite 1 (CBERS-1) and other ancillary data, and the overall accuracy for land cover classification was greater than 92.9% [23,24,25]. In our study, only original 6 first level land-use types of the datasets were used, namely cropland, woodland, grassland, water body, built-up land and unused land (Figure 2). 

In order to analyze the driving factors, ancillary data were used, such as census data obtained from the Jiangsu Statistics Bureau, including economic and population data. Previous studies have shown that land use changes are driven by various factors. In the present paper, we selected built-up land (BL) expansion to represent the land use change. Among these driving factors, population change and economic development were acknowledged as the main land use change drivers [11,37,40,51,52,53,54,55,56,57]. Due to the dual social structure of China, there are very significant differences between rural and urban areas, including aspects such as infrastructure, education and health care. In another words, people residing in urban areas, especially developed cities, may have a higher living standard. The gap leads to a mass population migration from undeveloped areas to developed areas, such as rural to urban areas. Meanwhile, population migration is complicated due to the special population policy in China. Therefore, we selected resident population (RP), urban population (UP) and population density (POD) as the demographic factors (Table 1). 

**Table 1:** Description of the selected driving factors of land use change.

Driven factor	Description
RP	People residing in a single location for more than 12 months; represents population pressure (unit: 10^4^)
UP	People residing in urban area for more than 12 months; represents urbanization level (unit: 10^4^)
POD	Resident population per unit area; represents population pressure (unit: /km^2^)
GDP	Represents economic development level (unit: 10^9^ yuan)
SGDP	Represents economic development and industrialization level (unit: 10^9^ yuan)
TGDP	Represents economic industrialization level (unit: 10^9^ yuan)
PerGDP	Represents economic development level (unit: yuan)
FAI	Represents capabilityof fixed assets investment (unit: 10^9^ yuan)
FI	Represents capability of improve living conditions of urban residents (unit: yuan)
UI	Represents capability of improving living conditions of farmers (unit: yuan)

The reform and opening up policy promoted a rapid economic development, particularly in Jiangsu Province. During 2000–2008, the average annual growth rate of gross domestic product (GDP) in Jiangsu was about 17.2%, increasing from 8,440.57 billion yuan to 29,915.38 billion yuan. Along with continuous economic development, governments will have more funds to improve living conditions and infrastructures, such as houses and roads [19,34,58,59,60], which will require more built-up land. Several indicators were selected to represent economic development, including GDP, secondary GDP (SGDP), tertiary GDP (TGDP), GDP *per capita* (PerGDP), fixed assets investment (FAI), *per capita* income of urban residents (UI) and *per capita* income of rural residents (FI) (Table 1). These statistical data were obtained from the Jiangsu Statistics Bureau at the prefecture-level.

### 2.3. Land Use Dynamics

The annual change rate can reveal the general trend of land use change, and in order to evaluate the land use change during the study period, the following formula was used to calculate the annual change rate:

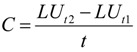
(1)
where *C* is the annual change rate of a given land use type; *LU*_*t*1_ and *LU*_*t*2_ are the area of the land at the times *t*1 and *t*2; and *t* is the interval of the calculation period years. *C* can show the general change trend of land use, and to further calculate the spatial distribution of LUCC intensity, we used sampling blocks (5 × 5 km) to intersect with the land use map, and input the major land use type information into each grid. Finally, the grid-based change intensities were calculated.

### 2.4. Spatial Pattern Changes

Land use changes will lead to structural changes in the landscape, which may affect the sustainable development of the ecosystem [46]. Landscape metrics have been widely used to describe the structures and pattern of a landscape [47,48]. As the area has been calculated in a previous section, another four indices were considered at the class level, which can describe the fragmentation, area distribution and shape complexity of the specific land use type: (1) number of patches (NumP); (2) mean patch size (MPS); (3) patch size standard deviation (PSSD); and (4) mean shape index (MSI). The metrics were calculated by using Patch Analyst 5.0 for ArcGIS 9.3.

### 2.5. Driving Force Analysis

Due to the fact that a correlation may exist among the selected variables, principal component analysis (PCA) and general linear model (GLM) were employed to identify the relative importance of the driving factors. In order to facilitate direct comparison, all variables were normalized before analysis. PCA was performed to reduce multicollinearity among variables and to generate new independent variables, referred to as principal components (PCs) [49]. The PCs can be identified by eigenvalues, such as eigenvalues larger than 1 or the inflection points of the scree plot of eigenvalues. In general, the PCs can represent the original variables when the cumulative percentage of the variances is within 85%. GLM was performed to generate the regression equation when the PCs were identified [50], then the PCs were replaced with the original variables, and finally the importances of the driving factors were determined. Statistical analyses were conducted in R 3.0.2.

## 3. Results and Discussion

### 3.1. Land Use Change

**Figure 3:**
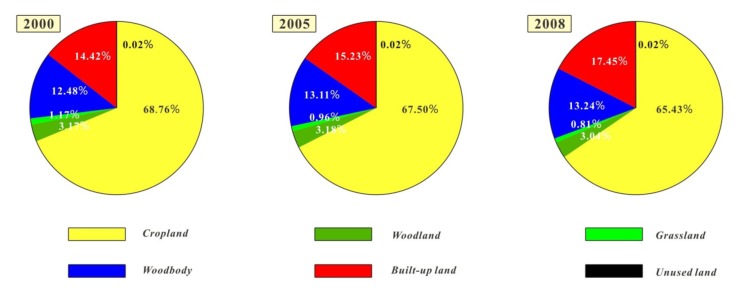
Land use change in Jiangsu during 2000–2008.

Figure 3 shows the landscape composition of Jiangsu Province from 2000 to 2008. As can be seen from the figure, cropland was the most dominant land use type, accounting for over 65.43% of the landscape in 2008, especially in central and northern Jiangsu, with 72.68% of the region in 2008. The second major land use type was built-up land, covering about 17.45% of the entire area in 2008, and around the city center it exhibited an obvious aggregation effect. The third major land use type was water body with 13.24% of the landscape, as Jiangsu has many lakes, such as Tai Lake in Suzhou, Hongze Lake in Huai’an, and Gaoyou Lake in Yangzhou. There were only small amounts of woodland, grassland and unused land in the study area, with woodland and grassland covering 3.04% and 0.81% in 2008, and unused land only representing 0.02% of the total land, thus in this paper we do not describe their changes. In general, land use changed significantly from 2000 to 2008, with cropland, woodland, grassland and unused land showing a sharp decline. In contrast, water body and built-up land exhibited increases, and these trends have continued and accelerated. 

As shown in Table 2, during 2000–2005, cropland decreased by 1.8% from 6,908,638 ha in 2000 to 6,784,445 ha in 2005, at a rate of 24,838.6 ha/year. Cropland was mainly converted to built-up land and water bodies. Meanwhile, there were 54,486 ha converted from built-up land and water bodies, and this phenomenon may be caused by land management policies, for example, the policy stating that temporary built-up land which occupied the basic farmland must be consolidated into cropland. Woodlands showed a small increase, which was primarily converted from cropland. At the same time, 1,265 ha was replaced by built-up land. Grassland shrank significantly, declining by 21.3%, from 117,197 to 96,750 ha, at a rate of 4,089.4 ha/year, which was mainly converted to water bodies. Water bodies increased from 125,369 to 1,315,574 ha, at an annual growth rate of 12,389 ha/year. New water bodies were mainly converted from cropland and grassland, at about 61,228 and 16,066 ha, respectively. Built-up land increased from 1,448,410 to 1,529,652 ha, with an average annual growth rate of about 16,248.4 ha, occupying a total of about 119,333 ha of cropland. 

**Table 2:** Conversion matrix of land use from 2000 to 2005 (unit: ha).

2000	2005						
	Cropland	Woodland	Grassland	Water body	Built-up land	Unused land	Total area
Cropland	6,722,875	4,757	313	61,228	119,333	132	6,908,638
Woodland	2,822	313,618	43	356	1,265	17	318,121
Grassland	4,259	114	94,950	16,066	1,807	1	117,197
Water body	12,531	230	1,211	1,233,936	5,715	6	1,253,629
Built-up land	41,955	835	232	3,851	1,401,531	6	1,448,410
Unused land	3	26	1	137	1	1,630	1,798
Total	6,784,445	319,580	96,750	1,315,574	1,529,652	1,792	10,047,793

During 2005–2008, land use change accelerated (Table 3). Cropland lost 198,622 ha during 2005–2008 at a speed of 66,207.3 ha/year, which was faster than the period of 2000–2005, and about 217,841 ha of the change region was occupied by built-up land. Woodland decreased from 319,580 to 305,692 ha at a rate of 4,629.3 ha/year, which was mainly replaced by cropland and built-up land, at about 11,585 ha and 6,026 ha, respectively. Grassland showed a continuous decline, with an annual change rate of −5,031.3 ha/year. Water body change growth rate slowed down, increasing 5,984 ha during the study period, at a rate of 1,994.7 ha/year, and was mainly converted from cropland. Built-up land exhibited a dramatic expansion and increased 221,747 ha, among which 217,841 ha was converted from cropland with an annual growth rate of up to 73,915.7 ha/year. 

**Table 3:** Conversion matrix of land use from 2005 to 2008 (unit: ha).

2005	2008						
	Cropland	Woodland	Grassland	Water Body	Built-Up Land	Unused Land	Total Area
Cropland	6,550,984	2,842	777	11,966	217,841	35	6,784,445
Woodland	11,585	301,778	2	165	6,026	24	319,580
Grassland	5,766	83	80,111	5,559	5,231	-	96,750
Water body	3,562	108	756	1,302,762	8,386	-	1,315,574
Built-up land	13,924	821	10	1,106	1,513,754	37	1,529,652
Unused land	2	60	-	-	161	1,569	1,792
Total area	6,585,823	305,692	81,656	1,321,558	1,751,399	1,665	10,047,793

Note: Nodata are expressed with a “-”.

**Figure 4:**
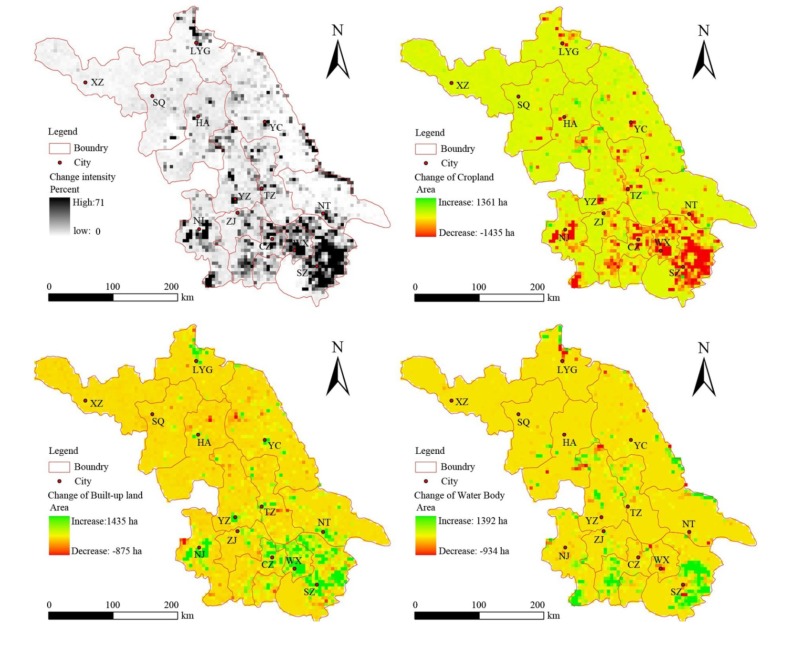
Spatial distribution of land use change from 2000 to 2005.

**Figure 5:**
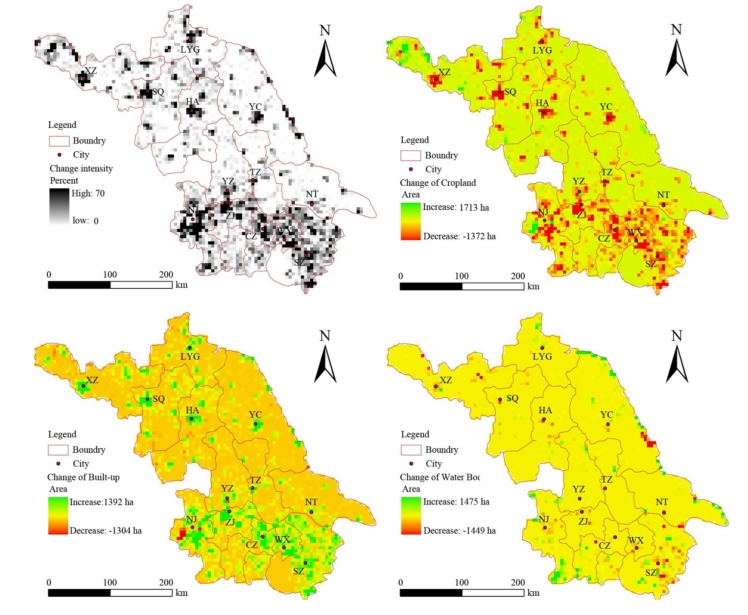
Spatial distribution of land use change from 2005 to 2008.

It can be seen from Table 2 and Table 3 that the main processes of land use change were the conversion between cropland, built-up land and water bodies. In order to assess the spatial distribution of the change, we mapped the change intensity from the land use datasets (Figure 4 and Figure 5). During 2000–2005, the area of land use change region was about 279,253 ha, and the annual change rate was 55,850.6 ha. As observed, the spatial difference was quite significant. Southern Jiangsu was more severe than central and northern Jiangsu, with an area of land use change of 163,482 ha, about 58.54% of the total change region. The hot spots of the change were mainly distributed throughout Suzhou, Wuxi, Changzhou and Nanjing. The primary characteristics were cropland loss, built-up increase and water body increase. In contrast, cropland increase mainly occurred in northern Jiangsu. 

During 2005–2008, land use change accelerated, with the area of land use changed region reaching 296,835 ha, at a rate of 98,945 ha/year. As can be seen from Figure 5, more hot spots began to appear in northern Jiangsu, and the major contribution of the change was the conversion of cropland to built-up land. The most remarkable characteristic was that the conversion was scattered closely around the city center. Built-up land and water bodies in southern Jiangsu increased continuously, but the growth rate slowed down. The highest loss of built-up land was observed in Nanjing, while hot spots of water body loss were located in Suzhou and Yancheng.

Along with the area change, spatial patterns also synchronize change. Table 4 shows the landscape metrics in 2000, 2005 and 2008. During the study period, the landscape metrics of each land use type showed different change trend. Cropland: the NumP experienced a decreased then increased process, while the area of cropland showed a continuous significant decline; the MPS and PSSD showed an increase then decline trend, and the MSI showed a continuous increase. These demonstrate that cropland become more fragmented and irregular in shape. Woodland: the NumP showed the same trend of the area, which experienced a continuous decline, whereas the MPS showed continuous increase. The PSSD experienced an increase then decreased process and the MSI showed little change. Grassland: the NumP and MSI experienced a decrease then increase process, while the area showed continuous decrease; the MPS and PSSD showed continuous decrease. Water body: the NumP showed continuous decrease along with the area increase, while the MPS, PSSD and MSI showed an increase trend. Built-up land: the changes of NumP, MPS and PSSD was the same as water body, while the area showed a continuous increase; the MSI experienced a decrease then increase trend. Unused land: the NumP showed continuous decrease along with the area loss; the MPS, PSSD and MSI displayed little change. 

**Table 4:** Landscape metrics change of land use in 2000, 2005 and 2008.

Land use type	NumP			MPS (ha)			PSSD			MSI		
	2000	2005	2008	2000	2005	2008	2000	2005	2008	2000	2005	2008
1	6,008	4,795	5,222	1,149.91	1,414.90	1,261.17	47,973.6	52,481.7	48,878.3	1.427	1.487	1.501
2	3,835	3,587	3,393	82.95	89.09	90.09	552.1	573.2	539.8	1.461	1.460	1.461
3	1,245	1,124	1,154	94.13	86.08	70.76	569.2	478.0	414.8	1.510	1.513	1.498
4	14,398	13,828	13,590	87.07	95.14	97.24	4,650.5	4,757.5	4,839.6	1.385	1.391	1.391
5	66,921	61,471	58,193	21.64	24.78	30.92	203.9	264.6	365.5	1.379	1.375	1.380
6	118	116	100	15.24	15.45	16.65	31.5	29.7	31.4	1.364	1.364	1.365

Note: 1, cropland; 2, woodland; 3, grassland; 4, water body; 5, built-up land; 6, unused land.

### 3.2. Driving Factors

#### 3.2.1. Socio-Economic Development

**Figure 6:**
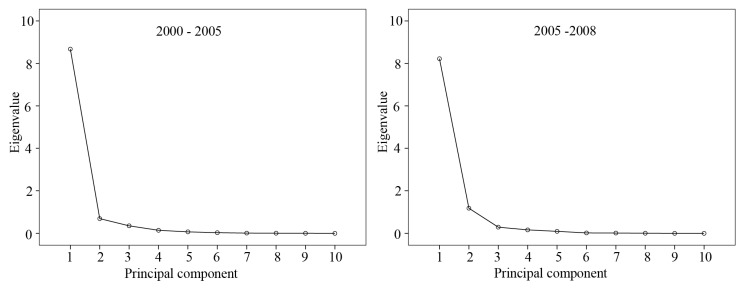
Scree plot of eigenvalues.

As shown in Table 5 and Table 6, there are high correlations between the BL and driving factors, as well as among driving factors. Therefore, the PCA was performed to reduce multicollinearity, and Figure 6 showed the scree plot of the eigenvalue of the PCA. According to the scree plot, PC1 and PC2 were selected as the regression variables. Then the parameters were estimated in GLM by maximum likelihood, and significance was accepted at *p* = 0.05 (Table 7).

**Table 5:** Correlations among variables for the period of 2000–2005.

Factor	BL	RP	UP	POD	GDP	SGDP	TGDP	PerGDP	FAI	UI	FI
BL	1										
RP	0.847 ^**^	1									
UP	0.830^**^	0.645 ^**^	1								
POD	0.762 ^**^	0.967 ^**^	0.574 ^**^	1							
GDP	0.967 ^**^	0.864 ^**^	0.858 ^**^	0.785 ^**^	1						
SGDP	0.975 ^**^	0.814 ^**^	0.840 ^**^	0.721 ^**^	0.987 ^**^	1					
TGDP	0.879 ^**^	0.914 ^**^	0.810 ^**^	0.872 ^**^	0.946 ^**^	0.883 ^**^	1				
PerGDP	0.980 ^**^	0.882 ^**^	0.710 ^**^	0.858 ^**^	0.940 ^**^	0.932 ^**^	0.896 ^**^	1			
FAI	0.876 ^**^	0.964 ^**^	0.759 ^**^	0.925 ^**^	0.919 ^**^	0.859 ^**^	0.971 ^**^	0.886 ^**^	1		
UI	0.796 ^**^	0.889 ^**^	0.633 ^*^	0.913 ^**^	0.846 ^**^	0.802 ^**^	0.883 ^**^	0.937 ^**^	0.893 ^**^	1	
FI	0.837 ^**^	0.833 ^**^	0.605 ^*^	0.848 ^**^	0.838 ^**^	0.833 ^**^	0.800 ^**^	0.943 ^**^	0.825 ^**^	0.957 ^**^	1

Note: ** *p* = 0.01; * *p* = 0.05.

**Table 6:** Correlations among variables for the period of 2005–2008.

Factor	BL	RP	UP	POD	GDP	SGDP	TGDP	PerGDP	FAI	UI	FI
BL	1										
RP	0.905 ^**^	1									
UP	0.847 ^**^	0.880 ^**^	1								
POD	0.890 ^**^	0.974 ^**^	0.828 ^**^	1							
GDP	0.824 ^**^	0.918 ^**^	0.886 ^**^	0.906 ^**^	1						
SGDP	0.786 ^**^	0.891 ^**^	0.840 ^**^	0.869 ^**^	0.992 ^**^	1					
TGDP	0.851 ^**^	0.938 ^**^	0.918 ^**^	0.940 ^**^	0.987 ^**^	0.959 ^**^	1				
PerGDP	0.520	0.712 ^**^	0.481	0.780 ^**^	0.768 ^**^	0.772 ^**^	0.761 ^**^	1			
FAI	0.674 ^*^	0.694^**^	0.875 ^**^	0.698 ^**^	0.814 ^**^	0.778 ^**^	0.828 ^**^	0.433	1		
UI	0.653 ^*^	0.804 ^**^	0.608 ^*^	0.847 ^**^	0.759 ^**^	0.735 ^**^	0.789 ^**^	0.915 ^**^	0.428	1	
FI	0.631 ^*^	0.768 ^**^	0.533	0.844 ^**^	0.782 ^**^	0.776 ^**^	0.787 ^**^	0.972 ^**^	0.475	0.942 ^**^	1

Note: ** *p* = 0.01; * *p* = 0.05.

**Table 7:** Results by performed GLM.

Period	β_PC1_	β_PC2_	*P*_PC1_	*P*_PC2_	DE (%)
2000–2005	0.932	0.219	0.00	0.04	91.60
2005–2008	0.841	-	0.00	-	81.04

Note: Nodata are expressed with a “-”.

Table 8 shows the results after replacing the PCs with the original variables. It is clear that both population change and economic development play very important roles in land use change, especially during the period of 2000–2005, during which the DE reached as high as 91.6%. This signifies that the land dynamics in Jiangsu Province can be largely explained by social-economic development. However, our discussion in this paper only considers driving force at the prefecture scale. As shown in Figure 4 and Figure 5, a significant regional difference in land use change is present. According to previous studies, the characteristics of land dynamics and their drivers usually changed at different spatial scales [60,61]. For example, there is a high rate of deforestation measured on the global scale, while forest expansion is observed in most developed countries [61]. Meanwhile, the same driver may play an opposite role in different places. For example, the studies conducted by Priess *et al.* [62] in Indonesia and Kidane *et al.* [63] in Ethiopia found a positive and significant correlation between cropland expansion and population growth. However, in the present study, we found there was a remarkable negative relationship between cropland change and population growth, and similar results were observed by Rayburn *et al.* [64] in the U.S. Midwest and Twumasii *et al.* [65] in Central Mississippi. In addition, Homewood *et al.* [66] found that population growth has little effect on land dynamics in semiarid Africa. 

**Table 8:** Coefficients of the driving factors.

Period	RP	UP	PoD	GDP	SGDP	TGDP	PerGDP	FAI	UI	FI
2000–2005	0.247	0.396	0.200	0.365	0.366	0.326	0.295	0.297	0.235	0.234
2005–2008	0.440	0.341	0.399	0.232	0.194	0.283	0.130	0.074	0.328	0.197

The relative importance of each factor was quite different during the different periods (Table 8). During 2000–2005, the most important driven factor was UP, followed by SGDP and GDP. During 2005–2008, the relative importance of each driven factor changed remarkably, especially the FAI, the importance of which declined significantly, and the most important driven factor was RP, followed by POD and UP. It indicates that time scale may be a key issue in the study of land use change. In brief, drivers of land use change may vary significantly along with social-economic development. This observation is consistent with conclusions reached by Hazell *et al.* [67]. Therefore, future research on land use change, particularly in simulating land dynamics should be done more carefully. Furthermore, the DE decreased to 81.04% during 2005–2008, which signifies that there were other important factors leading to the expansion of built-up land, such as policies. 

#### 3.2.2. Policies

The government’s policies can affect land use change [30,44], especially in China, which faces increasing serious food security and ecological environment problems, includes water pollution [42], land degradation [43], soil contamination and biodiversity loss [44,45]. Generally speaking, the government puts forward policies to promote economic development. Along with the economic growth, more land is required for infrastructural, industrial and residential purposes (Table 1 and Table 2). During 2000–2005, built-up land expansion of southern Jiangsu was more rapid than central and northern Jiangsu, at respective annual growth rates of 14,040, 2,047 and 1,161 ha/year. Since 2005, the Jiangsu government has enhanced the cooperation of the northern and southern areas, and one of the most important measures was the industry transfer from southern to northern Jiangsu. The effects of the policies were quite evident; for example, the built-up land increasing was noticeably accelerated compared to period 2000–2005 in northern Jiangsu, annual growth rates of central and northern Jiangsu were up to 7,856 and 26,210 ha/year. Rapid built-up land expansion may lead to other problems, such as food security and flooding (especially the 1998 Yangtze flood). In these cases, the central and provincial government will propose corresponding measures, such as during 2000–2008, water body increased by 67,929 ha, most of the new water body was distributed in southern Jiangsu (Figure 4), which was primarily caused by the policy of “reverting cropland to lake” (many water bodies were converted to cropland since 1960 in China. It was caused by the policy of “grain must be taken as the key link in agriculture” and it was known as land reclamation from lake (*wei hu zao tian*)) [30]. Meanwhile, most of the built-up land expansions were performed at the expense of cropland loss. In order to maintain food security, the central government performed a series of policy measures to protect cropland, especially basic cropland, such as the “Protection Rules of Basic Cropland” and maintain a “Dynamic Equilibrium of Cropland”. Although the policies cannot reverse the trend of cropland loss, they can slow its rate. As a consequence of the policy, about 96,409 ha of newly cropland was converted from other land use types during 2000–2008, and most of the new cropland was converted from built-up land. This was very different from other regions, such as in the Bale Mountains, Ethiopia [63] and Nairobi, Kenya [68], where, as pointed out by Mundia *et al.* [68], cropland was mainly converted from natural vegetation.

## 4. Conclusions

This study explored the spatial pattern of LUCC in Jiangsu Province, and the results indicated that the study area was primarily dominated by cropland, followed by built-up land and water bodies, which together accounted for over 95.7% of the total land, and the analysis also showed that the overall land use change was significant and characterized by continuous cropland loss and built-up land expansion, the cropland loss was mainly due to increasing land use for built-up land; in addition, the trend was accelerated and there were significant regional differences.

During 2000–2005, there was 185,763 ha cropland converted to other land use type, 119,333 ha (64.2%) converted to built-up land, and 61,228 ha (33.0%) converted to water bodies. There was a clearly uneven distribution of cropland loss or built-up land expansion, as most of the decreased cropland or built-up land expansion took place in southern Jiangsu, and the annual rate of built-up land expansion of southern Jiangsu was 6.4 times that of central Jiangsu and 11.2 times that of northern Jiangsu. During 2005–2008, cropland loss accelerated compared to 2000–2005, with about 233,461 ha of cropland converted to other land use types, and more than 93.3% converted to built-up land. Built-up land expansion was significant in all areas, especially in northern Jiangsu, where the annual growth rate was 22.58 times that of 2000–2005, the spatial distribution was relatively even compared to preceding period, as the annual rate of built-up land expansion of southern Jiangsu was 5.0 times of central Jiangsu and 1.4 times of northern Jiangsu. Furthermore, the spatial pattern changed along with the area, especially the pattern of cropland, which was quite different from the other land use types. The NumP of cropland experienced a decline then increase process, while other land use types showed decline trends; in addition, the MPS and PSSD were much larger than the others, and their shape were more irregular than others. 

The changes were caused by many factors, some of which may be related to or interact with each other, thus it was very difficult to accurately evaluate the contribution of each factor. Therefore, we analyzed the driven factors by PCA and GLM. The results showed that population growth and economic development had always played important roles in land use change. However, the relative importance of each driven factor changed with time. During the period of 2000–2005, the most important driven factor was UP, followed by SGDP and GDP, and RP become the most important factor during 2005–2008. It should be noted than not only may the relative importance of a specific driving factor vary, but the driven factors may as well. As shown in Table 7, the DE declined from 91.60% to 81.04%. This signifies that there were other driven factors affecting land use change during the period of 2005–2008. It means that we should more carefully when simulating land use change. In this paper, we analyzed the impact of policies on land use change, and it is observed that policies had significant impacts on land use change, which can be divided into direct and indirect impacts. Development policies usually had indirect impacts, particularly economic development policies, which promote the economic development to cause land use change, while land management policies had direct impacts. This signifies that governments should think comprehensively and cautiously when proposing a new policy.
